# Percutaneous Epidural Neuroplasty for Symptomatic Lumbar Juxtafacet Cysts

**DOI:** 10.3390/medicina60071042

**Published:** 2024-06-25

**Authors:** Juneyoung Heo, Hyung-Ki Park, Ji-Hoon Baek, Hye-Sun Ahn, Su-Chan Lee

**Affiliations:** 1 Joint & Arthritis Research, Department of Neurosurgery, Himchan Hospital, Seoul 21337, Republic of Korea; juneyoungheo@gmail.com; 2 Department of Neurosurgery, Soonchunhyang University College of Medicine, Seoul 31538, Republic of Korea; schnsphk@gmail.com; 3 Joint & Arthritis Research, Department of Orthopaedic Surgery, Himchan Hospital, Seoul 07999, Republic of Korea; jihoon011@naver.com (J.-H.B.); ahs0614@naver.com (H.-S.A.)

**Keywords:** juxtafacet, cyst, synovial, neuroplasty, lumbar, non-surgical, spinal stenosis

## Abstract

*Background and Objectives:* The term “Juxtafacet cyst” refers to both synovial cysts and ganglion pseudocysts associated with the lumbar facet joint. As conservative treatment for the juxtafacet cyst has a minimal effect, complete excision through surgery is considered the first choice of treatment. In this study, we retrospectively reviewed the clinical outcomes of percutaneous epidural neuroplasty for symptomatic lumbar juxtafacet cysts. *Materials and Methods:* We conducted a retrospective review of 34 patients with symptomatic juxtafacet cysts who visited a single institute from January 2010 to September 2023. Patients who received conservative treatment for at least 6 weeks but experienced no or insufficient effects were eligible for this study. After neuroplasty, a medical history check and neurological examination were performed during follow-up at 2 weeks, 1 month, 2 months, 3 months, 6 months, and once a year thereafter. *Results:* The pain improved for all patients to a VAS score of 3 or less immediately after neuroplasty; however, four of those patients (11%) had pain that worsened eventually to the same level as before the procedure and required surgery. The results showed that, regardless of cyst size, in cases with severe stenosis of the spinal canal, the outcome of neuroplasty was poor and often eventually required surgery. The cyst size was not associated with the procedure results. In addition, if the cyst was present at the L4–L5 level, or if diabetes mellitus was present, the likelihood of future surgery was significant (*p*-value = 0.003). *Conclusions*: Percutaneous neuroplasty showed a better success rate than other non-surgical treatments. In addition, severe spinal stenosis (Schizas grade C or higher), L4–L5 level, or diabetes mellitus produced a high possibility of surgery due to recurrence.

## 1. Introduction

A juxtafacet cyst includes both synovial cysts and ganglion pseudocysts associated with the lumbar facet joint [1,2]. Despite histopathological differences, true synovial cysts and pseudocysts do not differ in clinical features, treatment, or prognosis [3,4].

Various cysts can form within the lumbar spinal canal such as perineural cysts, dermoid cysts, arachnoid cysts, parasitic cysts, and infectious cysts [5]. Among them, cysts associated with the lumbar facet joint have various names (e.g., synovial cyst, ganglion cyst, pseudocyst, lumbar intraspinal cyst, cyst of the ligament flavum) because their origin, pathogenesis, and histology are diverse and unclear. In 1974, Kao CC et al. grouped the synovial cyst and ganglion pseudocyst together and termed the group juxtafacet cysts [1].

Based on histological results, a synovial cyst can be assumed to be associated with the synovial membrane of the facet joint, and a ganglion pseudocyst associated with the ligament flavum. Because clinical presentation, treatment, and outcome do not differ between these occurrences, these two cysts have been suggested as different endpoints of the same spinal degenerative process [3]. In 2017, Chebib I et al. histologically evaluated 75 resected specimens termed “synovial cysts” and “lumbar cysts” and found a large portion not derived from the synovial line but a pseudocystic degenerative change in the ligament flavum [6]. This finding indicates that juxtafacet cysts share a pathogenesis with spinal stenosis.

Juxtafacet cysts are thought to be associated with spinal degeneration, and the rate of observation using MRI is 0.8–2.0%. The symptoms are similar to those of lumbar disc herniation and vary depending on the area and extent of nerve compression. These cysts rarely resolve spontaneously [7]. As conservative treatment for juxtafacet cysts has a minimal effect, complete excision through surgery is the first choice of treatment. However, attempts at non-surgical treatment are ongoing, with percutaneous cyst puncture or steroid injection being common methods.

We hypothesized that percutaneous epidural neuroplasty would be the most effective non-surgical treatment method, with the injection of steroids into the juxtafacet cyst surface being effective at removing adhesion around the cyst. We have been treating juxtafacet cysts for several years with the non-surgical treatment method of percutaneous epidural neuroplasty. The purpose of this study was to determine how the imaging characteristics of the spinal joints and other clinical and radiological factors affect the treatment results of percutaneous epidural neuroplasty for symptomatic lumbar juxtafacet cysts.

## 2. Materials and Methods

We conducted a retrospective review of patients with symptomatic juxtafacet cysts who visited a single institute from January 2010 to September 2023. All patients underwent a medical history check, a neurological examination, and imaging (lumbar roentgenography, CT, MRI). Patients with symptoms (radiating pain, low back pain) for more than 1 month and corresponding juxtafacet cyst lesions on imaging tests were selected and administered conservative treatment such as non-steroidal anti-inflammatory drugs (NSAIDs) and physical therapy. The patients who did not experience any improvement were treated with transforaminal epidural steroid injection at least twice. Patients who received this conservative treatment for at least 6 weeks but experienced no or insufficient effects were eligible for this study. A basic laboratory examination was performed before neuroplasty and after the correction of abnormal coagulation. For patients taking an antiplatelet, the procedure was performed after discontinuation for a corresponding period of time depending on the type of drug.

Patient symptoms were classified as (1) radiculopathy with lumbago, (2) radiculopathy only, and (3) lumbago only; the degree of pain was measured using the visual analog scale (VAS). After the procedure, a medical history check and neurological examination were performed during follow-up at 2 weeks, 1 month, 2 months, 3 months, 6 months, and once a year thereafter. If there were no specific symptoms, a radiologic examination was not performed. The location and size of the juxtafacet cyst were confirmed on MRI, and the maximal diameter was measured on MRI T2-weighted transverse view. Only cases in which the cyst was attached to the facet joint or in the adjacent ligament flavum were included in this study. The exclusion criteria for the study were a rapidly worsening weakness on neurological examination, neurologic deficit corresponding to cauda equina syndrome, accompanying vertebral fracture, pregnancy, or diseases that may seriously affect the outcome of the procedure such as malignant tumor.

Six neurosurgeons with more than 5 years of neuroplasty experience at a single institute performed the procedures using same procedural protocol.

### Percutaneous Epidural Neuroplasty Procedure

All patients underwent neuroplasty using the caudal approach. We used a steerable navigation catheter (NaviCath^®^, Myelotec Co., Ltd., Hanam, Republic of Korea). The patient was placed in a prone position with the hip slightly flexed so the coccyx tip could be easily palpated. After inserting the trocar and neuroplasty catheter through the sacral hiatus using lateral fluoroscopy, the position was confirmed using anterior–posterior and lateral fluoroscopy and the catheter tip was adjusted to the periphery of the lesion. In this study, the cysts of all patients were located on the posterolateral side of the spinal canal, connected to the joint facet. After placing the catheter tip at the cyst location of each patient identified on MRI, 3 mL of contrast agent (Omnipaque, GE Healthcare Korea, Seoul, Republic of Korea) was administered through the catheter to assess the epidural space and pre-adhesiolysis status. After identifying areas around the lesion where contrast was poor due to adhesion with epidurography, mechanical adhesiolysis was performed using zigzag movements of the catheter tip, and then epidurography was performed to confirm the contrast was available in the area of the filling defect. Next, 4 mL of 40% triamcinolone acetate and 6 mL of 0.2% ropivacaine containing 1500 units of hyaluronidase were administered to the lesion site, and contrast elimination was confirmed. Hypertonic saline was not administered during neuroplasty under local anesthesia because it may cause severe pain.

## 3. Results

A total of 57 patients visited the hospital with a symptomatic juxtafacet cyst that persisted for more than 1 month. A total of 34 patients, excluding 23 patients whose symptoms improved with conservative treatment including NSAIDs, physical therapy, and two or more transforaminal epidural steroid injections for 6 weeks, were included in the present study. There were 19 females and 15 males, and the average age at the time of the procedure was 66 ± 11.09 years (45–85 years). Among the subjects, 15 (44%) had hypertension, 7 (20.6%) diabetes mellitus, and 12 (35.3%) were taking an antiplatelet or anticoagulant (Table 1). The average duration of symptoms before neuroplasty was 11 months (1–60 months), and the average follow-up period after the procedure was 37.5 months (2–66 months). The most frequently involved level was L4–L5 in 29 patients (85%), followed by L5–S1 in 5 patients (15%). The most common symptom was radiculopathy with lumbago, observed in 27 patients (79%), followed by only lumbago in 6 patients (17%) and only radiculopathy in 1 patient (2%). Radiculopathy was unilateral in all cases. One patient with radiculopathy had motor weakness (chronic, mild ankle weakness).

**Figure 1:**
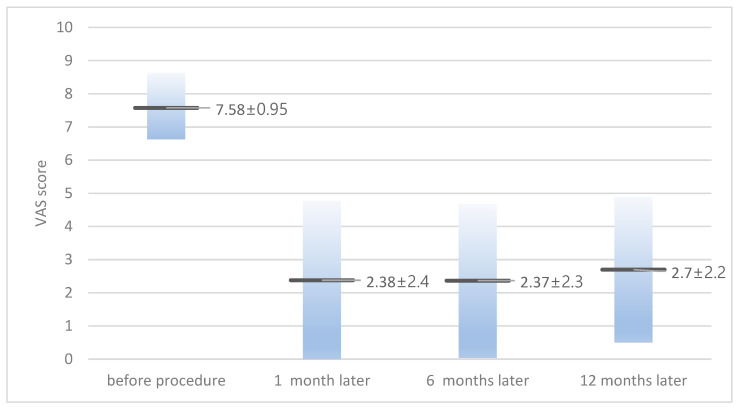
Visual analog scale (VAS) score of patients before and after neuroplasty.

The average VAS score before the procedure was 7.58 ± 0.95 (6–10), and for those at 1 month, 6 months, and 12 months after the procedure it was 2.38 ± 2.4, 2.37 ± 2.3, and 2.7 ± 2.2, respectively (Figure 1). When classified based on symptoms, in patients with radiculopathy with or without lumbago, the VAS score decreased from 7.6 ± 0.99 before the procedure to 2.5 ± 2.7 at 1 month after the procedure. In patients with lumbago only, the VAS score decreased from 7.5 ± 0.83 before the procedure to 1.6 ± 0.81 at 1 month after the procedure. There were 11 (32%) cases where symptoms recurred during the follow-up period, and all experienced relapses within 12 weeks. Among the relapses, four patients (11.7%) complained of symptoms of the same severity as before the procedure and underwent surgical treatment (laminectomy and cyst removal). Among the 28 patients with radiculopathy, 10 (35%) experienced a recurrence of symptoms and 4 (14%) progressed to surgery. All four patients who underwent surgery complained of radiculopathy with lumbago at the time of initial visit, and none of the patients with lumbago as a symptom progressed to surgery. The remaining seven patients who did not undergo surgery underwent conservative treatment including epidural steroid injection, oral NSAIDs, and physical therapy, and their VAS score was maintained at an average of 3.2 ± 0.75 (2–4) during the follow-up period. The average juxtafacet cyst size was 6.35 ± 2.49 mm (2–11.8 mm); for the size in patients who underwent surgery, it was 5.82 ± 2.36 mm.

## 4. Discussion

In the present study, all patients experienced an improvement in pain (VAS score of 3 or less) immediately after neuroplasty. However, four patients (11%) eventually experienced pain that worsened to the same level as before the procedure and required surgery. For the remaining 30 patients (88%), symptoms gradually improved until 1 month after neuroplasty. After that, however, symptoms slightly worsened in some patients over the next 6 months and then plateaued in most. A total of 7 patients (20%) remained in a tolerable state after the procedure, requiring additional conservative treatment such as epidural steroid injection, and the remaining 23 patients (67%) had mild pain but did not require additional treatment. During the average follow-up period of 37.5 months, the average VAS score was 2.48 ± 2.75 and most patients reported an occasional use of pain medication. In a large systematic evaluation of fluoroscopically guided steroid injection with cystic puncture by J.F. Martha et al. (2009), most patients, regardless of surgery, required the regular use of pain medication, indicating a degree of pain chronicity in juxtafacet cysts [8].

Patients with lumbago-only symptoms (six patients) had better results that lasted longer than patients with radiculopathy, and none progressed to surgery. Among the remaining 28 patients and excluding lumbago-only patients, 4 patients (14%) progressed to surgery, 7 patients (25%) required additional conservative treatment, and 17 patients (60%) did not require additional treatment.

**Figure 2:**
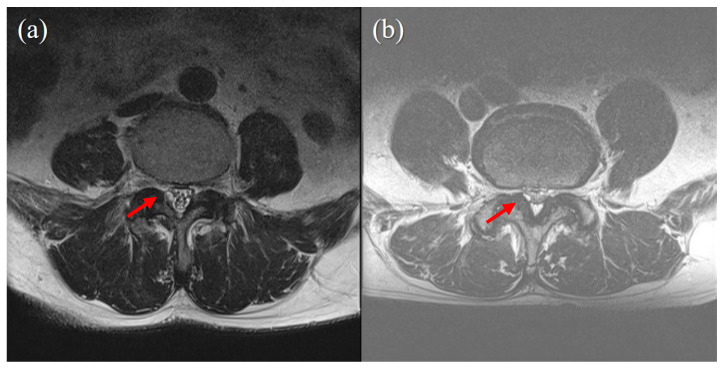
Cyst size does not correlate with success of percutaneous epidural neuroplasty. (**a**) case of successful treatment with percutaneous epidural neuroplasty. A 3 mm-sized juxtafacet cyst on the Rt posterolateral spinal canal connected to a facet joint. Ther was no visible CSF signal, and no rootlet was recognizable (Schizas grade C). (**b**) A case of failed percutaneous epidural neuroplasty, for which surgery was eventually performed. A 2 mm-sized juxtafacet cyst in the same location as (**a**). A CSF signal and rootlets were visible (Schizas grade B).

In the present study, we analyzed whether cyst size, pre-procedural VAS score, symptom duration before procedure, predisposing factors (hypertension, diabetes mellitus, antiplatelet or anticoagulant use), age, gender, and BMD were associated with procedure success. The degree of pain (VAS score) and duration before the procedure were not associated with the result of the procedure. The cyst size was also not associated with the procedure results (Figure 2). However, in the four patients who underwent surgery, the cyst size was smaller than in the patients who did not undergo surgery (5.82 mm vs. 6.42 mm).

Regardless of the cyst size, in cases where there was severe pressure on the cauda equina due to stenosis of the spinal canal, the outcome of neuroplasty was poor and often eventually led to surgery (*p*-value = 0.000). When the 4 patients who underwent surgery were compared with the 30 other patients, spinal stenosis was sufficiently severe to cause the obliteration of the CSF space, and posterior epidural fat on MRI corresponded to Grade C (no rootlets recognizable, no visible CSF signal but epidural fat present posteriorly) of the Schizas system [9]. Patients classified as Grade A or B had a significantly lower probability of undergoing surgery, and the probability that their symptoms would not worsen was significantly higher than in patients classified as Grade C (Table 2). The pathogenesis of a juxtafacet cyst is thought to be related to spinal degeneration caused by the motion of a spinal segment [10] and appears to be the final stage of the degenerative process of the lumbar spine [11]. One explanation for the failure of the neuroplasty of juxtafacet cysts in patients with severe spinal stenosis is because the disease occurs in the same or a consecutive segment as spinal stenosis. In addition, if the cyst was present at a level other than the L4–L5, the likelihood of surgery was significantly low (*p*-value = 0.003). Patients with diabetes were more likely to undergo surgery (*p*-value = 0.03; Table 3).

The reported probability of satisfactory results from percutaneous cyst puncture varies from 37% to 80%, with an average of 55%. The remaining 45% of patients ultimately undergo surgery due to a lack of improvement or recurrence [8,12,13,14,15]. In a study by Allen TL et al., 34% of patients who underwent fluoroscopic cyst puncture received surgery within 1 year [12]. In a study by Martha JF et al., percutaneous cyst puncture was performed in 55% of patients at an average of 8.4 months [8]. In a study by Slipman CW et al., 50% of patients who underwent percutaneous cyst puncture with steroid injection ultimately received surgery [16]. In most studies on percutaneous puncture treatment, the probability of symptom exacerbation that led to surgery was approximately 50%, and surgery was performed within 6–12 months after non-surgical treatment. In several recent studies, successful cystic rupture contributed to worsening symptoms in some cases [17,18] and more severe disability after 3 years of follow-up [8]. The inside of the synovial cyst is rich in prostaglandins, proteases, and cytokines [19]. Assumedly, when cyst puncture is performed, these internal inflammatory factors spread into the epidural space, causing radiculopathy or neurologic deficit.

There have been several studies in which epidural steroid injection was attempted as a conservative treatment for synovial cysts; however, in most patients, the effect was not maintained over time and ultimately led to surgery in many cases [16,20,21]. In those studies, all epidural steroid injections were transforaminal, which is likely because the drug can be administered closer to the lesion than with the interlaminar or caudal route. However, in the transforaminal route, the maximum possible insertion point for the needle is the entrance to the lumbar intervertebral foramen and is somewhat distant from the synovial cyst. Adhesion and scar tissue between the lesion and the needle prevent the steroid from moving to the lesion, and this space is further restricted when foraminal or central stenosis is present. The drug penetrates not only the lesion area, but also the surrounding area. Neuroplasty allows the catheter tip to directly access the lesion site while performing mechanical adhesiolysis, allowing the effective administration of steroids only to the lesion site. Therefore, even if the same amount of steroid is administered, the treatment effect may be better because the drug spread is lower to areas other than the lesion. Considering that percutaneous epidural neuroplasty and intra-articular steroid injection are more effective than other routes of steroid injection, it can be assumed that the effectiveness of the steroid application to the cyst will impact the treatment effect. However, the cyst size and location and the degree of pressure on the nerve roots have not been analyzed, preventing comparative studies with other non-surgical treatments.

When comparing the results of this study with those of studies using percutaneous puncture treatments, the degree of pain reduction was similar but the treatment success rate was higher. In a 12-month follow-up study after steroid injection into the intra-articular space, the probability of symptom improvement was about 75%, showing better results than cyst puncture [22,23]. Looking at these results, it was difficult to determine whether the cyst had ruptured. Rather, a positive effect of a steroid injection into the cyst is noted. In our study, while performing neuroplasty, mechanical adhesiolysis was performed as much as possible around the cyst, which may have caused the rupture or leakage of the cyst. However, because the inside of the cyst was not visualized and no changes in cyst size were observed, cyst rupture could not be confirmed. It is likely that effective steroid injection into the cyst surface would produce better results than cyst rupture and showed a more favorable outcome.

We speculated that neuroplasty would have better results than other non-surgical treatments because the adhesive and inflammatory nature of the juxtafacet cyst can be resolved. A lumbar synovial cyst can adhere throughout the spinal canal [24,25,26]. Because the cyst is well attached to the dura mater, recurrence or reoperation after surgery is possible. The larger the cyst size, the more severe the adhesion tends to be [26]. This adhesion can entrap the nerve and cause refractory back or lower extremity pain [27,28,29]. The adhesive nature of a lumbar juxtafacet cyst occurs due to an inflammatory response to the cyst and surrounding tissue. When the juxtafacet cyst is observed under an optic and light-polarized microscope, a synovial-type epithelium (synovial cyst) or ganglion cyst (containing fibrinoid or myxoid material) is visible, surrounded by mixed inflammatory cells. In addition, vascular proliferation as well as fibroblastic or myofibroblastic proliferation are visible due to an inflammatory reaction [6]. Such inflammatory changes are observed in specimens removed at the time of surgery after symptoms have occurred [30]. Apparently, the synovial lining is damaged by inflammation and replaced by other cells and tissues [6]. The more severe the inflammatory reactions are, the greater the damage to the synovial villi will be, resulting in scar tissue and calcification [31]. Assumedly, secondary changes such as inflammation occur in the cyst and are associated with the occurrence of symptoms [6,31].

The present study had a small number of patients and was conducted at a single institution, limiting the generalization of the results. It was also retrospective in nature and does not provide strong evidence to support selection bias and specific non-quantitative outcome measures (particularly an MRI-based classification system) in determining which patients will undergo these two procedures simultaneously. However, the results showed that the MRI-based grade for spinal stenosis rather than the juxtafacet cyst size can be an important factor in predicting the success of neuroplasty. In addition, the segment containing the cyst and the presence or absence of diabetes mellitus can also affect neuroplasty. However, because the data were based on a retrospective study, a definite conclusion could not be reached.

Our study is the first to apply neuroplasty as a non-surgical treatment for juxtafacet cysts. We hope that neuroplasty can be a sufficiently effective non-surgical treatment for juxtafacet cysts and can also be helpful in determining treatment direction.

## 5. Conclusions

Non-surgical treatment for symptomatic juxtafacet cysts is limited. In the present study, percutaneous neuroplasty was performed as treatment for symptomatic juxtafacet cysts and showed a better success rate than other non-surgical treatments. In addition, in the presence of Schizas Grade C or higher, L4–L5 level, or diabetes mellitus, there was a high possibility of surgery due to recurrence. However, because the data were based on a retrospective study, a definite conclusion could not be reached. Prospective studies are needed to further evaluate percutaneous neuroplasty for symptomatic juxtafacet cysts.

## Figures and Tables

**Table 1:** Demographic and procedural details of 34 patients who underwent percutaneous neuroplasty for symptomatic juxtafacet cyst.

	Data
Age	66 ± 11.09 years
Gender (male–female)	15:19
Height	160.7 ± 10.00 cm
Weight	64.82 ± 10.24 kg
BMI	25.03 ± 2.95 k/m^2^
Hypertension	Yes 19, No 15
Diabetes mellitus	Yes 7, No 27
Antiplatelet	Yes 12, No 22
VAS score before neuroplasty	7.58 ± 0.95 (6–10)
Average symptom duration before neuroplasty	11 months (1–60 months)
Juxtafacet cyst size	6.35 ± 2.49 mm
Affected level	
L4–L5	26 (4)
L5–S1	8 (0)

VAS: visual analog scale.

**Table 2:** Relationship of spinal stenosis grade, affected level, and diabetes mellitus in patients who received neuroplasty for symptomatic juxtafacet cyst.

	The 4 Patients Who Underwent Surgery	The 30 Patients Who Did Not Undergo Surgery	*p*-Value
Spinal stenosis grade (Schizas system)			
A	0	4	0.00
B	0	23	
C	4	7	
Affected level			
L4–L5	4	22	0.03
Others	0	8	
Diabetes mellitus			
Yes	3	4	0.03
No	1	26	

**Table 3:** Relationship between Schizas grade and success of neuroplasty.

Schizas Grade	Grade A	Grade B	Grade C	*p*-Value
4 patients who underwent surgery	0	0	4	0.00
30 patients who did not undergo surgery	4	23	3	
11 patients whose symptoms recurred during follow-up	0	7	4	0.05
23 patients who did not require additional treatment	4	16	23	

## Data Availability

The data presented in this study are available from the corresponding author upon request. The data are not publicly available due to privacy reasons.
